# Global, regional and national burden of pancreatic cancer and its attributable risk factors from 2019 to 2021, with projection to 2044

**DOI:** 10.3389/fonc.2024.1521788

**Published:** 2025-01-14

**Authors:** Xiao Li, Yi Zhang, Zeyi Yan, Wenkai Jiang, Shaozhen Rui

**Affiliations:** ^1^ The Second Clinical Medical School, Lanzhou University, Lanzhou, China; ^2^ The First School of Clinical Medicine, Lanzhou University, Lanzhou, China; ^3^ Department of General Surgery, The First Hospital of Lanzhou University, Lanzhou, China

**Keywords:** pancreatic cancer, risks, incidence, death, disability-adjusted life years

## Abstract

**Background:**

To estimate the global burden of pancreatic cancer in 2019 and 2021 including incidence, mortality, and disability-adjusted-life-years (DALYs).

**Methods:**

Data on pancreatic cancer incidence, mortality and DALYs were downloaded from the Global Health Data Exchange. The 95% uncertainty intervals (UIs) were reported for annual numbers and rates (per 100,000 populations).

**Results:**

In 2021, there were 508,532 (95% UI: 462,09 to 547,208) incident cases of pancreatic cancer globally, of which 273,617 (250,808 to 299,347; 53.8%) were in males. The age-standardized incidence rate was 6.0 (5.5 to 6.5) per 100,000 people in 2019 and decreased to 5.9 (5.4 to 6.4) per 100,000 people in 2021. There was a 3.9% increase in the number of deaths from pancreatic cancer from 486,869 (446,272 to 517,185) in 2019 to 505,752 (461,224 to 543,899) in 2021. There was a 3.5% increase in DALYs due to pancreatic cancer, increasing from 10.9 million (10.1 to 11.7) in 2019 to 11.3 million (10.5 to 12.2) in 2021. In 2021, the highest age-standardized death rates were observed in Greenland and Monaco, and the highest age-standardized DALY rates were observed in Greenland and Uruguay. The numbers of incident cases and deaths peaked at the ages of 70 to 74 years. The pancreatic cancer burden increased as the socio-demographic index increased. To 2044, the number of incident cases and deaths will be more than 875 thousand and 879 thousand, respectively.

**Conclusion:**

The disease burden of pancreatic cancer remains high, especially in high-income regions. More cancer prevention measures are needed in the future to reduce the burden of pancreatic cancer.

## 1 Introduction

Pancreatic cancer is one of the greatest challenges in oncology and public health. Globally, new incident cases of pancreatic cancer have significantly increased in the last two decades, and it is now the sixth most common cause of cancer-related deaths worldwide (1, 2). After surgical treatment, most pancreatic cancer patients experience tumor recurrence or metastasis. In most cases, pancreatic cancer patients need continuous chemotherapy and surveillance for recurrence and metastasis (3). These medical procedures demand high quality medical care as well as a large number of medical expenditures, highlighting the importance of the healthcare system for cancer management.

Previous studies have estimated the epidemiological trends and patterns of pancreatic cancer before 2019 (4, 5). Data on pancreatic cancer incidence and death were limited from 2019 to 2021, particularly when nuanced time and geographical locations were considered. Over the last 30 years, the Global Burden of Diseases (GBD) Study has generated multiple versions of worldwide estimates for various disease metrics (6). The GBD 2021 reported the global burden of 371 diseases and injuries and 88 risk factors, using a variety of data sources and novel statistical methods (7, 8). In this study, we evaluated the global burden of pancreatic cancer from 2019 to 2021 based on data from the GBD 2021, aiming to provide novel insight into cancer epidemiology and prevention.

## 2 Methods

### 2.1 Data sources

Epidemiological data, including total counts and age-standardized rates (ASRs) of incidence, mortality and disability-adjusted life years (DALYs), were downloaded from the GBD 2021. The GBD 2021 provided available data on 371 diseases and injuries by age sex, year and location (7, 8). The general methodology and data processing for the GBD 2021 have been detailed in some publications (7, 9). In this study, we gathered data in 2019, 2020 and 2021 from five socio-demographic index (SDI) quintiles, 21 GBD regions, all countries/territories and 17 age groups (15 to 19, 20 to 24, 25 to 29, ……, 95 plus). The identification of pancreatic cancer was based on the International Classification of Disease coding system 10th edition using the following codes: C25-C25.9 and Z85.07 (7).

### 2.2 SDI

The SDI is a composite measure used in GBD studies to reflect the social development of a region or country. The SDI value is calculated based on the total fertility rate of women under 25 years of age, the average education level of the population over 15 years, and the lag-distributed income per capita (10). The SDI ranges from 0 to 1; the higher the SDI value is, the better the social development of the region or country. The SDI values in the GBD dataset can be downloaded at https://ghdx.healthdata.org/record/global-burden-disease-study-2021-gbd-2021-socio-demographic-index-sdi-1950–2021.

### 2.3 Decomposition analysis

Decomposition analysis is a method used to assess the relative contributions to the change in the number of cases between two subgroups (11, 12). In this study, we performed decomposition analysis to identify the factors related to the alterations in the total number of DALYs from 2019 to 2021 as the product of three factors: (1) population size, (2) age structure of the population, and (3) prevalence of disease (13).

### 2.4 Risk factors

Smoking, high fasting plasma glucose (FPG) and high body-mass index (BMI) are risk factors included in the GBD 2021 for pancreatic cancer. The GBD 2021 defined current smokers as individuals who currently use any smoked tobacco product on a daily or occasional basis, and defined former smokers as individuals who quit using all smoked tobacco products for at least six months, where possible, or according to the definition used by the given survey (8). High FPG was defined as any level above the theoretical minimum-risk exposure level, which is 4.9 to 5.3 mmol/L (8). High BMI for adults is defined as BMI greater than 20 to 23 kg/m^2^ (8). The relative risk of pancreatic cancer was derived from published population-based data sources. Risk factor estimation in the GBD study was based on the comparative risk assessment framework established to compute risk factor estimates (8, 14).

### 2.5 Data analysis

Metrics were estimated as counts, age-specific rates and ASRs. The ASR was calculated based on the GBD standard population structure. All estimates are reported with 95% uncertainty intervals (UIs) (15).For changes over time, we present percentage changes from 2019 to 2021 (8). Correlations between the ASR and SDI were analyzed using a scatter diagram. The “Nordpred” package in R software has been shown to perform well in projecting cancer incidence and mortality trends (16, 17).We used “Nordpred” package to predict the pancreatic cancer incidence and deaths from 2022 to 2044. Data visualization was performed via R software (version 4.2.2).

## 3 Results

### 3.1 Global burden of pancreatic cancer overview

The global incidence of pancreatic cancer was 489,862 (95% UI: 447,390 to 520,122) in 2019, and increased to 508,533 (462,091 to 547,208) in 2021, representing a 3.8% increase in incidence over the last two years. The age-standardized incidence rate (ASIR) of pancreatic cancer per 100,000 people decreased from 6.04 (5.5 to 6.42) per 100,000 people in 2019 to 5.96 (5.39 to 6.42) per 100,000 people in 2021.

The number of deaths due to pancreatic cancer worldwide increased from 486,869 (446,272 to 517,185) in 2019 to 505,752 (461,224 to 543,899) in 2021. From 2019 to 2021, the age-standardized death rate (ASDR) decreased from 6.03 (5.5 to 6.41) per 100,000 people to 5.95 (5.4 to 6.41) per 100,000 people. The ASDRs attributable to smoking, high FPG and high BMI were 0.85, 1.59 and 0.1 per 100,000 people, respectively.

There were 10,936,307 (10,124,259 to 11,660,743) DALYs from pancreatic cancer worldwide in 2019, which increased to 11,316,963 (10,464,697 to 12,169,336) in 2021, representing a 3.5% increase in DALYs over the last two years. The age-standardized DALY rates decreased from 132.18 (122.01 to 140.88) per 100,000 people to 130.33 (120.52 to 140.13) per 100,000 people in 2021.

### 3.2 Spatial distribution of the pancreatic cancer burden

Estimates for incidence and death due to pancreatic cancer as absolute values and age-standardized rates per 100,000 people in 2019 and 2021 at the regional and national levels are presented in **Table 1** and **Supplementary Table 1**. In both 2019 and 2021, the highest ASDRs were observed in Greenland: 16.83 per 100,000 in 2019 and 15.89 per 100,000 in 2021. United Arab Emirates was the next leading country for the highest ASDR from pancreatic cancer in 2019 (16.7 per 100,000), and Monaco had the highest ASDR in 2021 (13.49 per 100,000). The ASIR followed a very similar pattern: it was highest in Greenland (16.09 per 100,000) and United Arab Emirates (15.57 per 100,000) in 2019 and highest in Greenland (15.21 per 100,000) and Monaco (13.27 per 100,000) in 2021. At the regional level, high-income Asia Pacific, high-income North America and Western Europe were the top three regions with the highest ASIRs in 2021, whereas Central Europe, high-income Asia Pacific and high-income North America were the three regions with the highest ASDRs in 2021.

**Table 1:** Age-standardized incidence and death rates of pancreatic cancer in 2019 and 2021 across 21 GBD regions.

GBD regions	ASIR in 2019	ASDR in 2019	ASIR in 2021	ASDR in 2021
Andean Latin America	5.38 (4.43 to 6.5)	5.81 (4.81 to 6.96)	5.17 (3.99 to 6.52)	5.56 (4.3 to 6.97)
Australasia	8.94 (8.08 to 9.54)	7.99 (7.24 to 8.5)	8.62 (7.77 to 9.23)	7.73 (6.97 to 8.24)
Caribbean	5.12 (4.71 to 5.62)	5.43 (4.99 to 5.94)	5.09 (4.5 to 5.75)	5.4 (4.76 to 6.11)
Central Asia	4.42 (4.03 to 4.81)	4.68 (4.27 to 5.1)	4.27 (3.77 to 4.77)	4.53 (4.01 to 5.08)
Central Europe	9.35 (8.84 to 9.74)	9.8 (9.26 to 10.22)	9.27 (8.49 to 10.04)	9.72 (8.91 to 10.52)
Central Latin America	4.45 (4.19 to 4.63)	4.75 (4.46 to 4.95)	4.54 (4.06 to 5.04)	4.83 (4.33 to 5.36)
Central Sub-Saharan Africa	2.22 (1.54 to 3.02)	2.4 (1.65 to 3.31)	2.27 (1.57 to 3.17)	2.46 (1.68 to 3.49)
East Asia	5.53 (4.59 to 6.58)	5.62 (4.68 to 6.68)	5.64 (4.56 to 6.8)	5.72 (4.63 to 6.87)
Eastern Europe	8.38 (8.01 to 8.75)	8.63 (8.24 to 9)	8.28 (7.65 to 8.98)	8.52 (7.88 to 9.23)
Eastern Sub-Saharan Africa	1.96 (1.64 to 2.48)	2.13 (1.78 to 2.71)	2.01 (1.66 to 2.53)	2.18 (1.8 to 2.75)
High-income Asia Pacific	10.87 (9.47 to 11.74)	9.72 (8.48 to 10.5)	10.69 (9.3 to 11.53)	9.56 (8.34 to 10.34)
High-income North America	10.24 (9.42 to 10.68)	9.36 (8.6 to 9.77)	10.2 (9.38 to 10.65)	9.32 (8.56 to 9.75)
North Africa and Middle East	4.52 (4.1 to 4.93)	4.69 (4.27 to 5.11)	4.52 (3.97 to 5.08)	4.69 (4.12 to 5.26)
Oceania	2.31 (1.89 to 2.91)	2.49 (2.03 to 3.16)	2.28 (1.86 to 2.89)	2.46 (2 to 3.12)
South Asia	1.39 (1.28 to 1.5)	1.49 (1.37 to 1.61)	1.41 (1.25 to 1.56)	1.51 (1.35 to 1.67)
Southeast Asia	3.29 (2.85 to 3.75)	3.48 (3.02 to 3.97)	3.33 (2.88 to 3.87)	3.53 (3.04 to 4.1)
Southern Latin America	9.36 (8.77 to 9.91)	9.95 (9.31 to 10.54)	8.61 (7.98 to 9.17)	9.13 (8.42 to 9.72)
Southern Sub-Saharan Africa	5.67 (5.11 to 6.22)	6.13 (5.52 to 6.73)	5.76 (5.09 to 6.36)	6.22 (5.49 to 6.86)
Tropical Latin America	5.93 (5.52 to 6.17)	6.37 (5.9 to 6.64)	5.86 (5.42 to 6.16)	6.27 (5.76 to 6.59)
Western Europe	10.02 (9.17 to 10.63)	9.69 (8.84 to 10.31)	9.54 (8.71 to 10.16)	9.26 (8.43 to 9.84)
Western Sub-Saharan Africa	1.88 (1.59 to 2.16)	2.05 (1.75 to 2.36)	1.92 (1.62 to 2.22)	2.09 (1.77 to 2.42)

GBD, Global Burden of Disease; ASIR, age-standardized incidence rate; ASDR, age-standardized death rate.

### 3.3 Burden of pancreatic cancer by age and sociodemographic development in 2021

**Figure 1:**
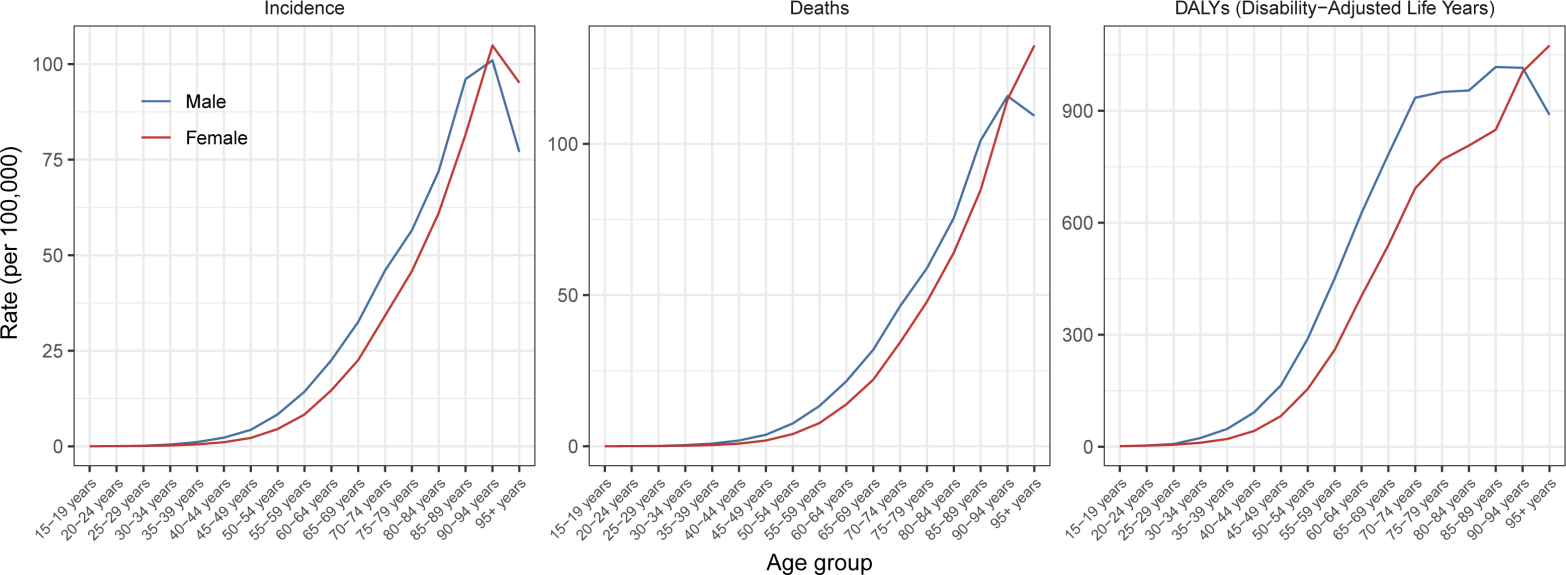
Global age-specific rates of incidence, mortality, and disability-adjusted-life-years of pancreatic cancer per 100,000 populations by sex in 2021.

The incidence and death rates for males peaked at the ages of 90 to 94 years, whereas the highest DALY rate was observed at the ages of 85 to 89 years in 2021. For females, the pancreatic cancer incidence rate peaked at the ages of 90 to 94 years, while death and DALY rates increased with increasing age. Additionally, until the ages of 90 to 94 years, males presented higher incidence, death, and DALY rates compared to females in the same age group (**Figure 1**). The incidence, death and DALY rates of pancreatic cancer in 2021 in different age groups by SDI are shown in **Supplementary Figure 1**.

**Figure 2:**
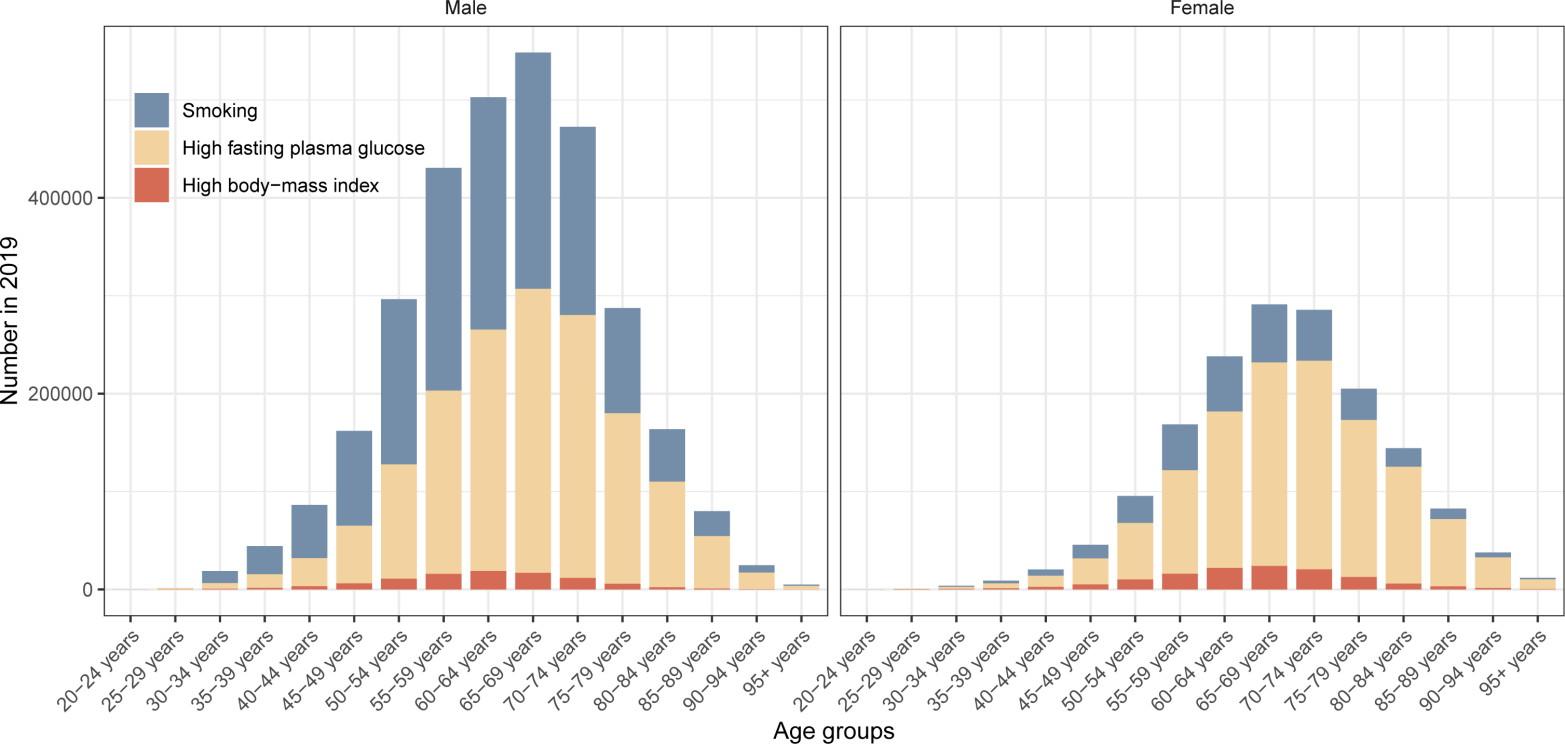
Global number of disability-adjusted-life-years of pancreatic cancer attributable to smoking, high fasting plasma glucose and high body-mass index in different age groups by sex in 2021.

**Figure 2** shows the distribution of global pancreatic cancer DALYs attributable to three risk factors across age groups in 2021. In 2021, males accounted for 3,124,369 pancreatic cancer DALYs attributable to smoking, high FPG and high BMI, and females accounted for 1,640,146 DALYs. In females, high FPG was the primary risk factor for DALYs in all age subgroups in 2021. Among males under the age of 60 to 64 years, smoking was the predominant risk factor for DALYs. The global age-specific DALY rates of pancreatic cancer attributable to three risk factors in 2021 are shown in **Supplementary Figure 2**.

**Figure 3:**
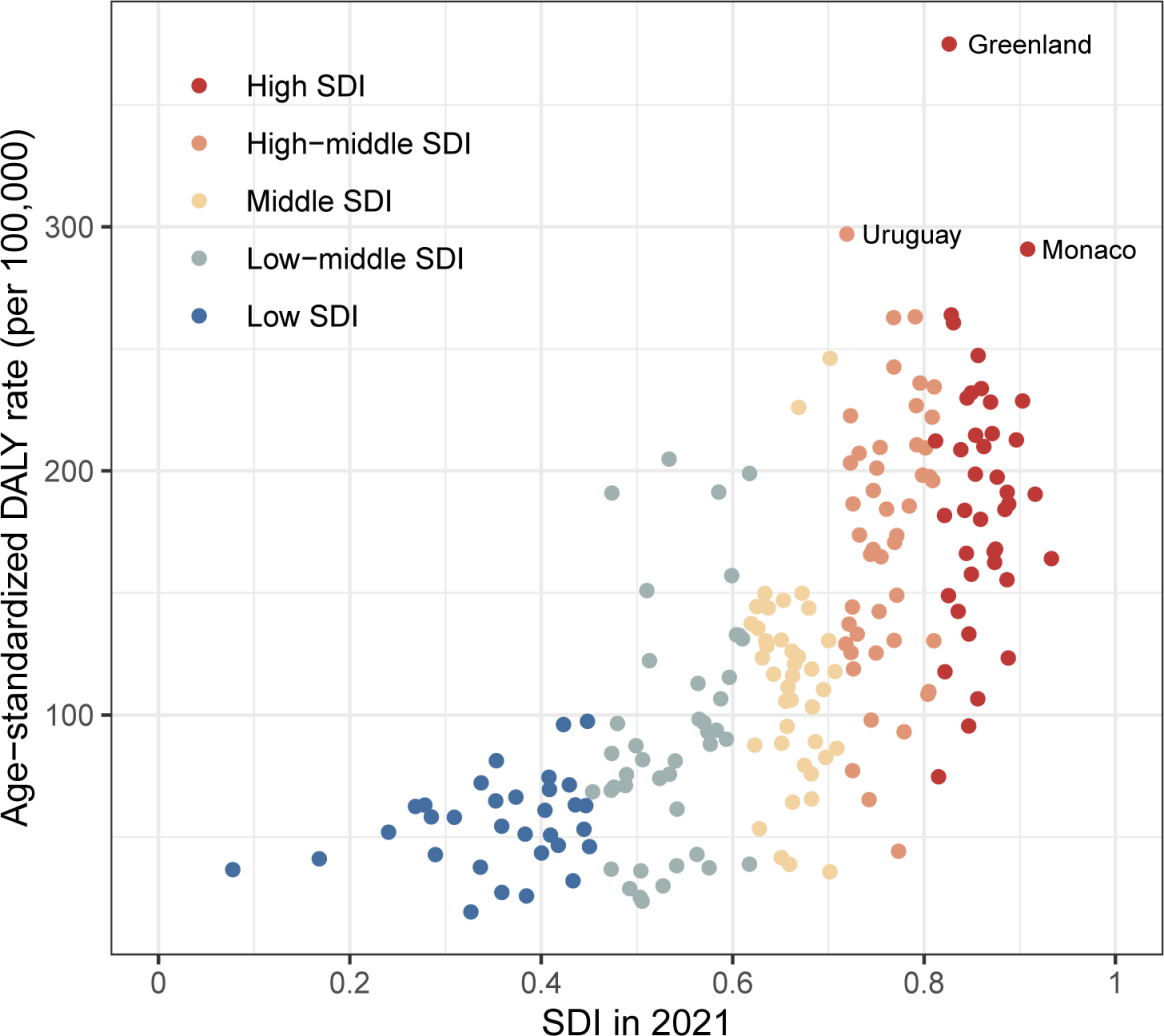
The relationship between age-standardized DALY rate of pancreatic cancer and SDI in all countries/territories in 2021 DALY: disability-adjusted-life-years; SDI: socio-demographic index.

Among the five SDI quintiles, the ASIR of pancreatic cancer ranged from 1.59 (1.33 to 1.9) per 100,000 in countries with low SDI to 10.0 (9.07 to 10.61) per 100,000 in countries with high SDI, and the ASDR of pancreatic cancer ranged from 1.73 (1.45 to 2.07) per 100,000 in countries with low SDI to 9.37 (8.52 to 9.96) per 100,000 in countries with high SDI (**Supplementary Figure 3**). The numbers of incident cases, deaths and DALYs of pancreatic cancer in 2021 by SDI was shown in **Supplementary Figure 4**. To investigate the correlation between pancreatic cancer burden and countries’ sociodemographic development, we examined the relationship between age-standardized DALY rates and the SDI (**Figure 3**). The findings indicated that countries with high SDI presented age-standardized DALY rates, whereas countries with low SDI displayed considerably lower age-standardized DALY rates. Similar results were shown between ASIR and ASDR and SDI (**Supplementary Figures 5**, **6**).

### 3.4 Decomposition analysis of pancreatic cancer DALYs

**Figure 4:**
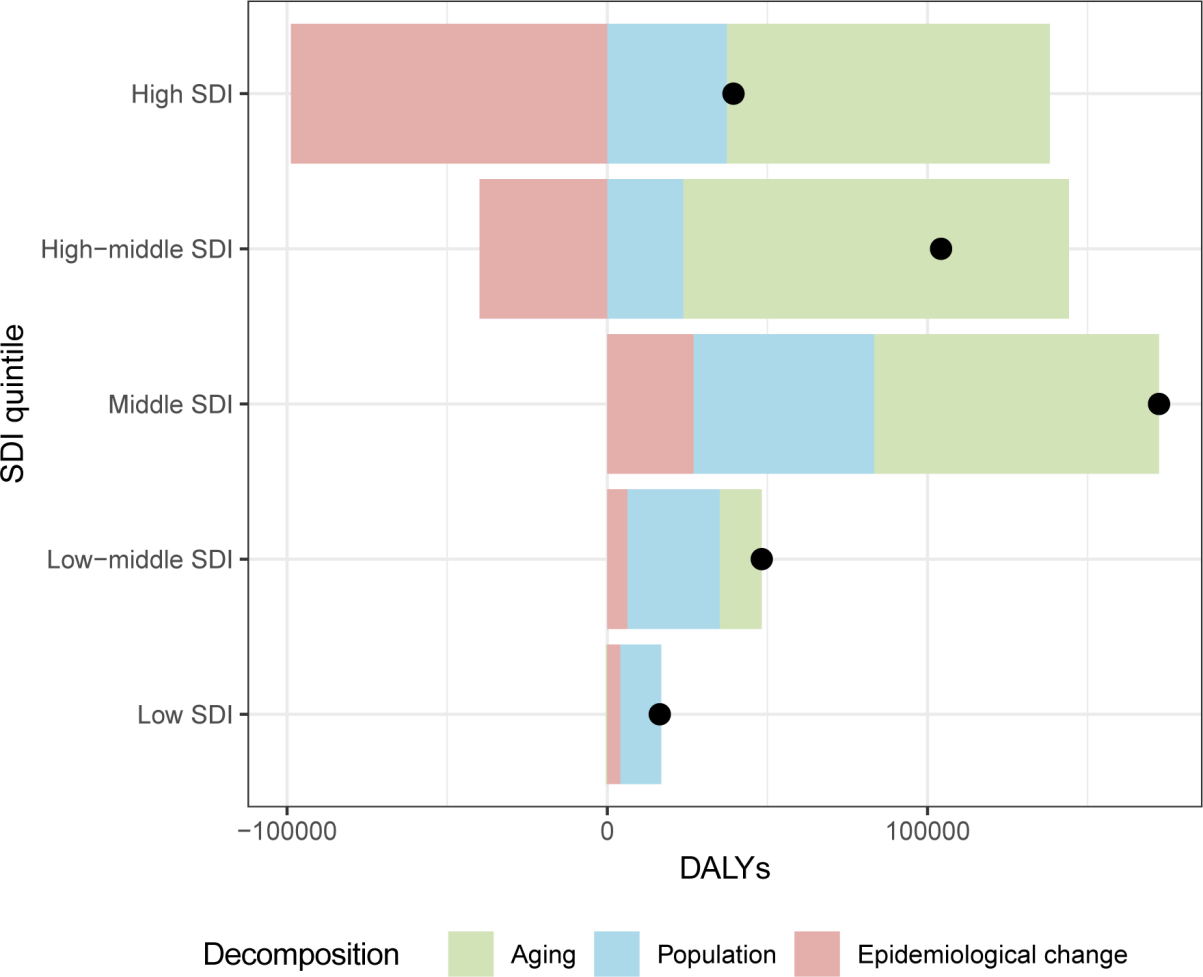
The decomposition analysis of pancreatic cancer DALYs change between 2019 and 2021 in different SDI quintiles. DALY: disability-adjusted-life-years; SDI: socio-demographic index.

In general, there was a noticeable rise in pancreatic cancer DALYs in the five SDI quintiles, with the most significant increase observed in the high-middle- and middle-SDI quintiles, which showed the greatest increase in total DALYs over the past two years (**Figure 4**). The contribution of aging to the overall difference in DALYs was greatest in the high-SDI quintile (255.92%), followed by the high-middle-SDI quintile (115.53%). Most of the increase in pancreatic cancer DALYs due to population growth was observed mainly in the high- (94.67%) and low-SDI quintiles (78.22%). The results of decomposition analysis of 21 GBD regions are shown in **Supplementary Figure 7**.

### 3.5 Predicted incident cases and deaths to 2044

**Figure 5:**
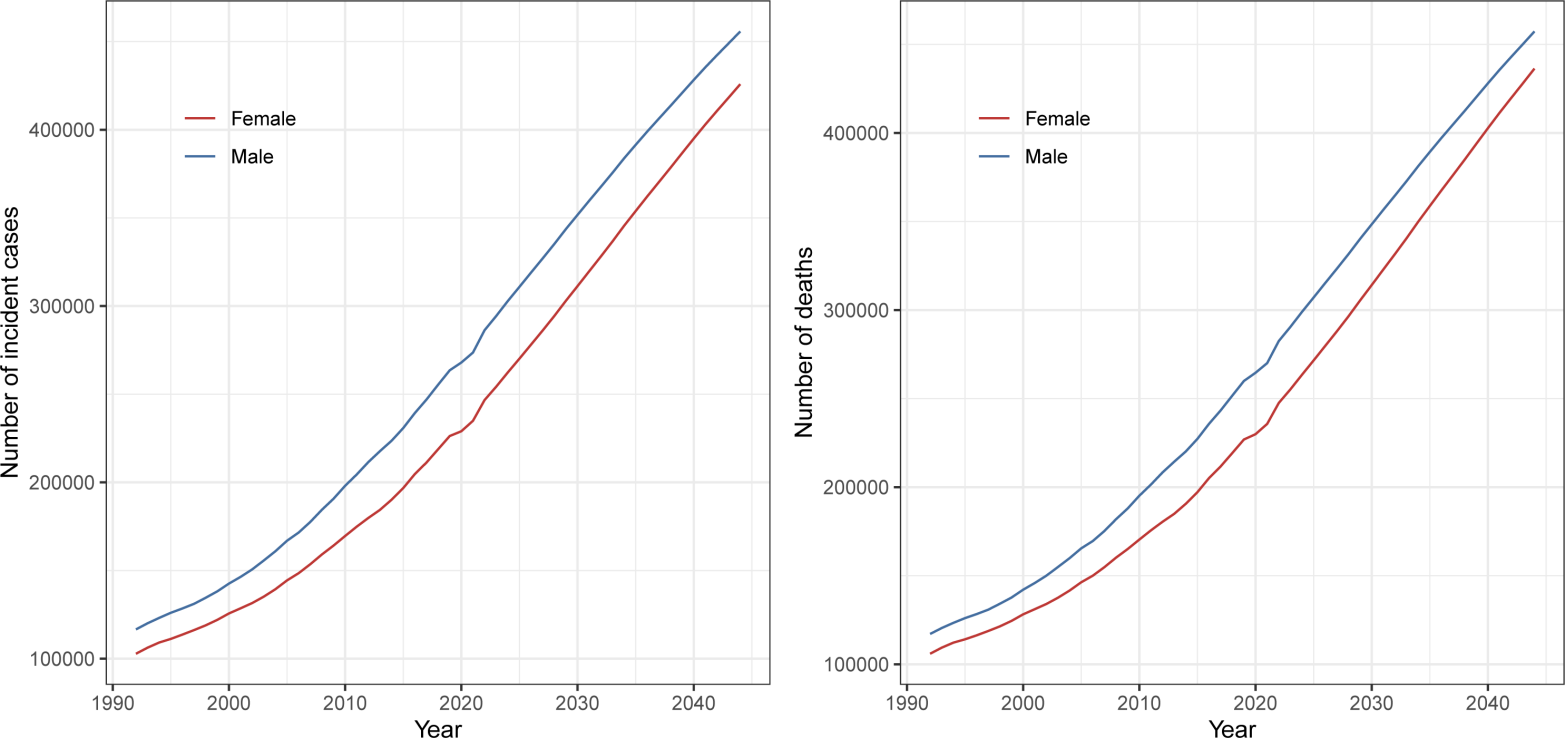
Global number of incident cases and deaths of pancreatic cancer from 1990 to 2021 and prediction to 2044 by sex.

It is predicted that the number of incident cases and deaths will continue to increase in the next 23 years (**Figure 5**). In 2044, the number of incident cases will be more than 455 thousand in males and more than 425 thousand in females. Moreover, the number of deaths will be more than 457 thousand in males and more than 436 thousand in females in 2044.

## 4 Discussion

Pancreatic cancer is a significant contributor to mortality rates globally, with a rising trend in both the number of incident cases and deaths. Cancer prevention is currently an important public issue worldwide. Our analysis revealed that the global pancreatic cancer burden remains high, especially in many high-income regions or countries; the age-standardized DALY rate increased with sociodemographic development. The substantial increase in pancreatic cancer DALYs suggests a change in population growth and aging populations, especially in high-middle and middle SDI countries. Under these circumstances, more effective cancer prevention methods and treatment strategies are needed to reduce the burden of pancreatic cancer.

In the last few decades, the global burden of pancreatic cancer has increased significantly. Global health goals are aimed at reducing the risk of and disability from noncommunicable diseases, including cancer: a 25% reduction in premature mortality by 2025 and a one-third reduction by 2030 (18). From 1990 to 2019, GBD analyses revealed worsening outcomes of cancer burden. However, GBD 2021 reports on a new trend: the age-standardized incidence, death and DALY rates of pancreatic cancer worldwide have slightly decreased in both 2020 and 2021. Although the number of years included is limited, this is an encouraging improvement in the progress made in the global pancreatic cancer epidemic over the past three decades.

However, preventing and treating pancreatic cancer remains a global challenge. Although pancreatic cancer ranks 12th in the number of cases of all cancers in 2022, it ranks 6th in the number of cancer deaths in 2022 (2). Over the last three years, the pancreatic cancer incidence and death rates have consistently increased in high-SDI regions. This pattern was similar to that reported in the previous GBD studies (4). Pancreatic cancer is a disease that occurs primarily in high-income countries (4). There is an association between socioeconomic factors and the pancreatic cancer diagnosis (19). One reason for the higher incidence of pancreatic cancer in high-income countries may be attributed to the complete cancer registry databases and advanced diagnostic techniques for detecting pancreatic cancer. Moreover, lifestyle factors also contribute to the high burden of pancreatic cancer in high-income countries. Smoking is a well-known important risk factor for pancreatic cancer. The overall risk of pancreatic cancer estimated for current and former smokers was 1.74 (95% confidence interval: 1.61 to 1.87) (20). From 1990 to 2019, there was a notable increase in the global number of smokers, growing from 0.99 billion to 1.14 billion (21). Smoking prevalence also varies widely between regions. It was reported that the prevalence of smoking among women in high-income regions is higher than in other regions (21). Obesity and diabetes are important metabolic factors associated with pancreatic cancer (22). In 2019, the highest age-standardized cancer mortality rate attributable to high fasting plasma glucose was highest in high-SDI regions and lowest in low-SDI regions (23). Similarly, prevalence of overweight and obesity were also higher in some countries in North America, Oceania and Western Europe (24). Since 1980, the prevalence of obesity has doubled in more than 70 countries (25). There were more than 400 thousand deaths and 11 million DALYs of cancer related to high BMI in 2019 (26). In 2021, there were 529 million people of all ages living with diabetes, yielding a prevalence of 6.1% worldwide (27). This change can be attributed to lifestyle changes in many developing countries. Dietary patterns tend toward a “Western dietary pattern”. This change includes increased intake of sugar, fat, and animal products, and reduced physical activity (28, 29). In developing regions, this “nutrition transition” has been abrupt, and it has led to a great increase in non-communicable diseases associated with diabetes and obesity, especially cancer.

The number of DALYs of pancreatic cancer has continued to increase since 2019, reflecting both the contribution of aging and population growth, especially in the middle-SDI region. The global number of cancer cases is anticipated to increase in the coming years, largely due to significant demographic shifts, including population growth and aging (30). The United Nations projects that the global population will reach 9.3 billion in 2050 and 10.1 billion in 2100 (31). Additionally, the global proportion of individuals aged 65 years and older increased from 6.1% in 1990 to 8.8% in 2017 (32). Aging and cancer share some overlapping features. Some features of aging, such as genomic instability, epigenetic changes, and chronic inflammation, are very similar to specific cancer features (33). We found that pancreatic cancer has a higher disease burden in the older population. The inactivation of genes in crucial senescence effector pathways may accelerate pancreatic carcinogenesis (34). Therefore, we need to pay more attention to pancreatic cancer screening in the elderly.

With the increasing population and population aging, the burden of pancreatic cancer is rapidly increasing with few recent improvements in patient survival. There is an increasing prevalence of diabetes and overweight in the population (35). Therefore, reducing exposure to risk factors may be an effective way to decrease the burden of pancreatic cancer. Healthcare systems should focus on reducing the prevalence of tobacco use, obesity and diabetes. Health care professionals should focus on the process and health of smoking cessation in the populations (36). The public health field should launch public welfare activities, such as healthy diet education and moderate exercise plans. On a population-wide scale, diet in low fat and sugar and regular exercise may be an effective prevention strategy that ultimately reduces the incidence of pancreatic cancer (37). Early screening for pancreatic cancer should be performed in populations at high risk. Individuals with multiple close family members affected by pancreatic cancer, those possessing a pathogenic variant in one of the genes associated with a high risk of pancreatic cancer, or people at high risk with pancreatic cysts history consider screening (38). There are already multiple clinical trials showing the role of new therapies for pancreatic cancer, and such explorations should continue in the future (39–41). Additionally, we should pay attention to cancer research equity. The theme of World Cancer Day 2024 was “Close the Gap”, highlighting the persistent disparities in cancer care (42). Geographical disparities in pancreatic cancer prevention and treatment quality between high-income and low-income countries must be addressed, to help those with the highest burden of cancer access necessary treatment.

There are several limitations to this analysis. First, data from GBD estimation have some limitations due to the lack of available and accurate data in some countries, especially in low-income countries. For countries/territories without epidemiological data, GBD estimation results are mostly based on modeling processes and trends from neighboring locations, leading to some uncertainty. Moreover, the lack of advanced cancer diagnostic tools and incomplete cancer registries in low-income countries may lead to underestimation of the pancreatic cancer burden. Third, our study included only three years, which is not able to obtain a definite change trend compared with previous GBD studies (from 1990 to 2017 or from 1990 to 2019), but we used the latest data from the GBD database (in 2020 and 2021). Future work should focus on collecting more available and accurate data on the epidemiology of pancreatic cancer, especially in developing countries. The population-based cancer registry should be expanded to cover more people. More attention should be given to the establishment and development of cancer registry databases in low-income regions and countries. We should also focus on the global economic burden of pancreatic cancer.

In 2020 and 2021, the burden of pancreatic cancer remained high in high-income countries, whereas it is increasing in low-income countries. More pancreatic cancer prevention and treatment strategies are needed not only to improve survival rates, but also to provide healthier and safer outcomes for a growing and aging population.

## Data Availability

Publicly available datasets were analyzed in this study. This data can be found here: https://vizhub.healthdata.org/gbd-results/.
