# Supplementary material for: Global, regional and national burden of pancreatic cancer and its attributable risk factors from 2019 to 2021, with projection to 2044
Source: Front Oncol. 2025 Jan 14;14:1521788. doi: 10.3389/fonc.2024.1521788 (PMC11772166; doi:10.3389/fonc.2024.1521788)
Supplement: Supplementary file 1 [file DataSheet1.docx]

**Supplementary figure 1**

Global age-specific rates of incidence, mortality, and disability-adjusted-life-years of pancreatic cancer per 100,000 population by SDI quintile in 2021. SDI: socio-demographic index

**
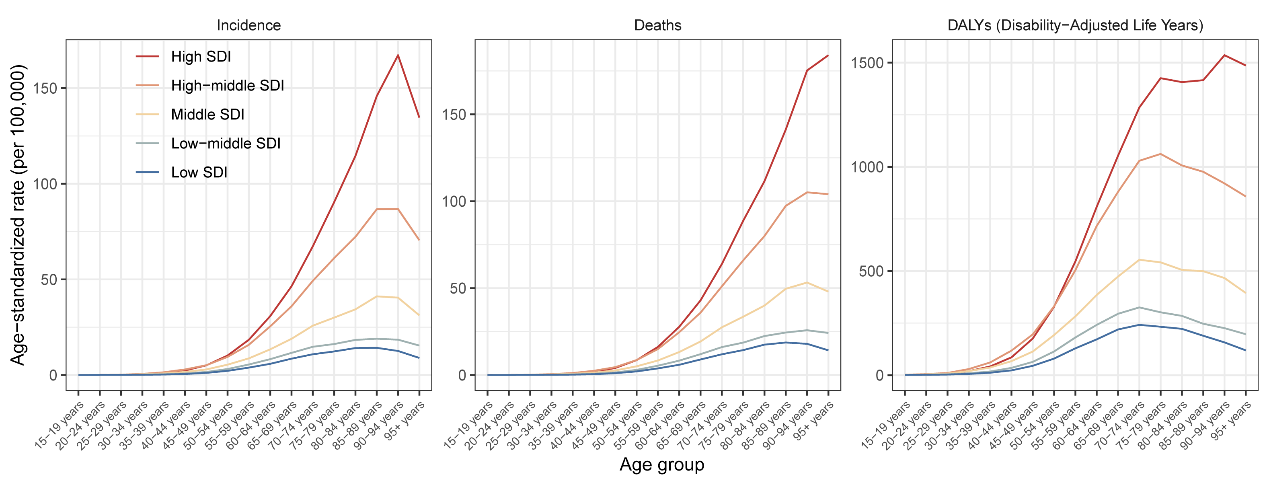
**

**Supplementary figure 2**

Global age-specific DALY rates of pancreatic cancer attributable to smoking, high fasting plasma glucose and high body-mass index per 100,000 population by SDI quintile in 2021. DALY: disability-adjusted-life-years; SDI: socio-demographic index

**
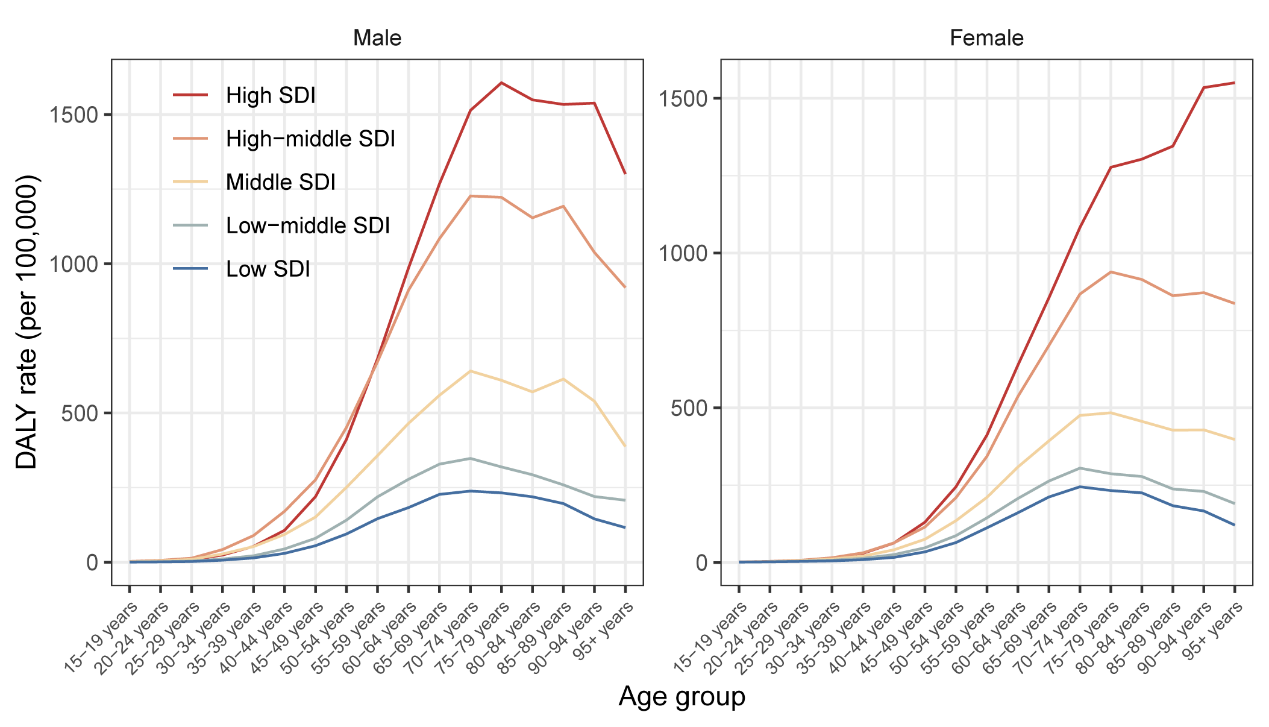
**

**Supplementary figure 3**

The age-standardized incidence, mortality, and disability-adjusted-life-year rate of pancreatic cancer in five SDI quintiles by sex in 2021. SDI: socio-demographic index

**
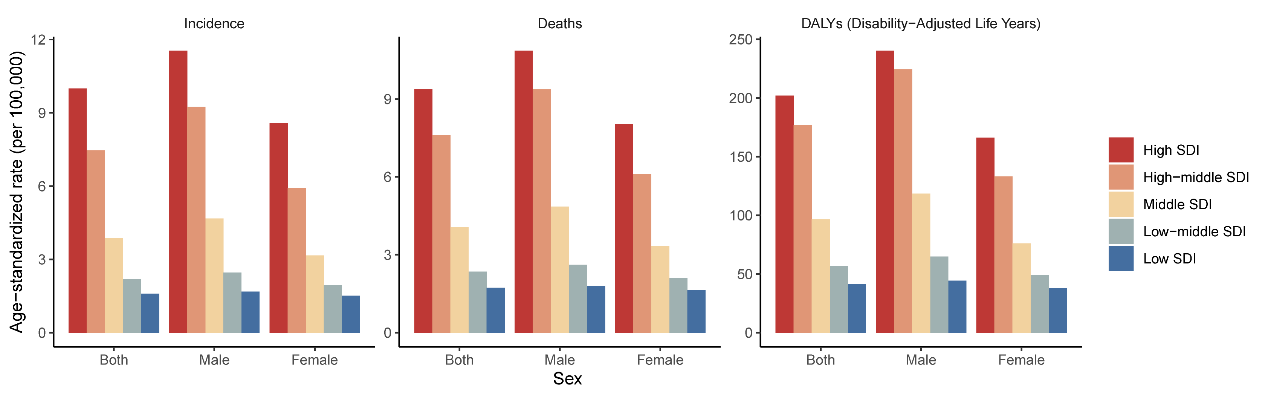
**

**Supplementary figure 4**

The number of incident cases, deaths, and disability-adjusted-life-years of pancreatic cancer in five SDI quintiles by sex in 2021. SDI: socio-demographic index

**
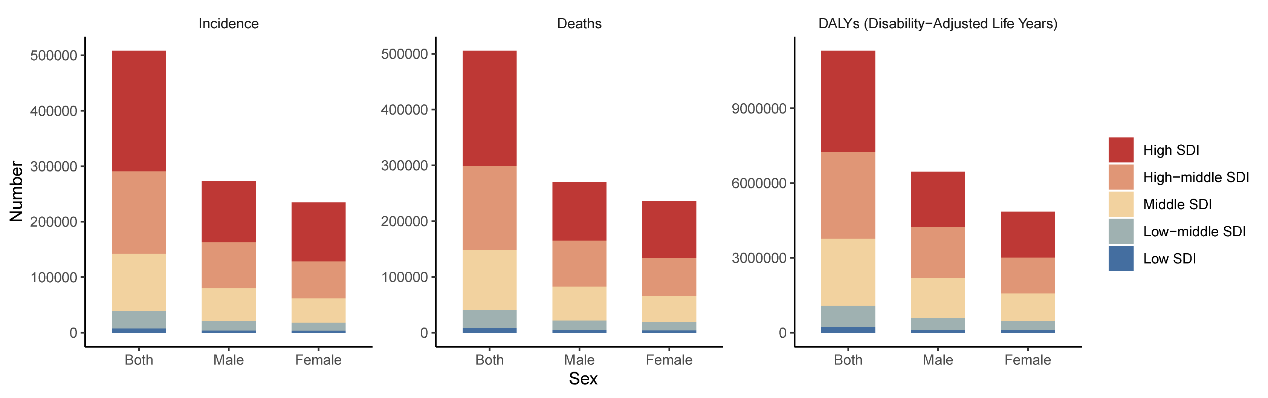
**

**Supplementary figure 5**

The relationship between age-standardized incidence rate of pancreatic cancer and SDI in all countries/territories in 2021. SDI: socio-demographic index

**
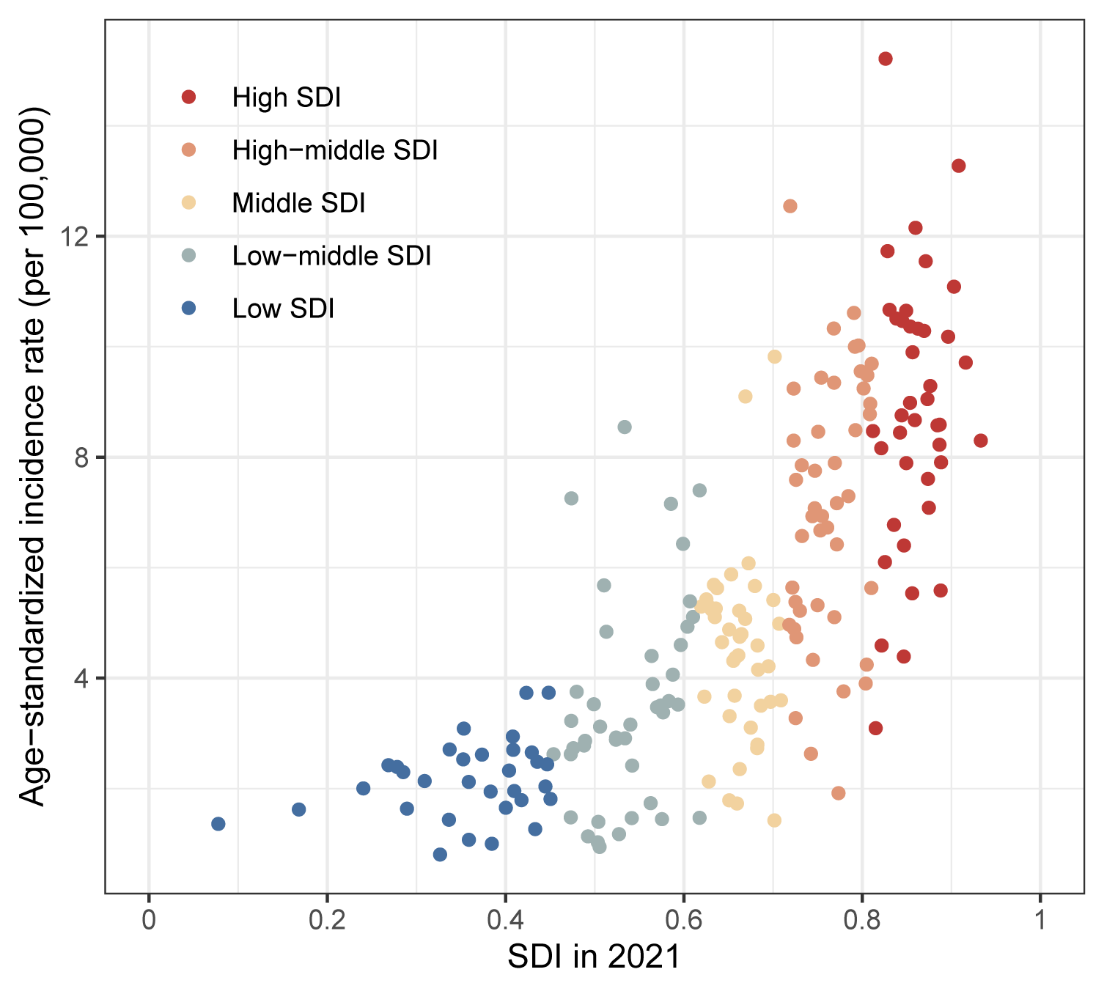
**

**Supplementary figure 6**

The relationship between age-standardized death rate of pancreatic cancer and SDI in all countries/territories in 2021. SDI: socio-demographic index

**
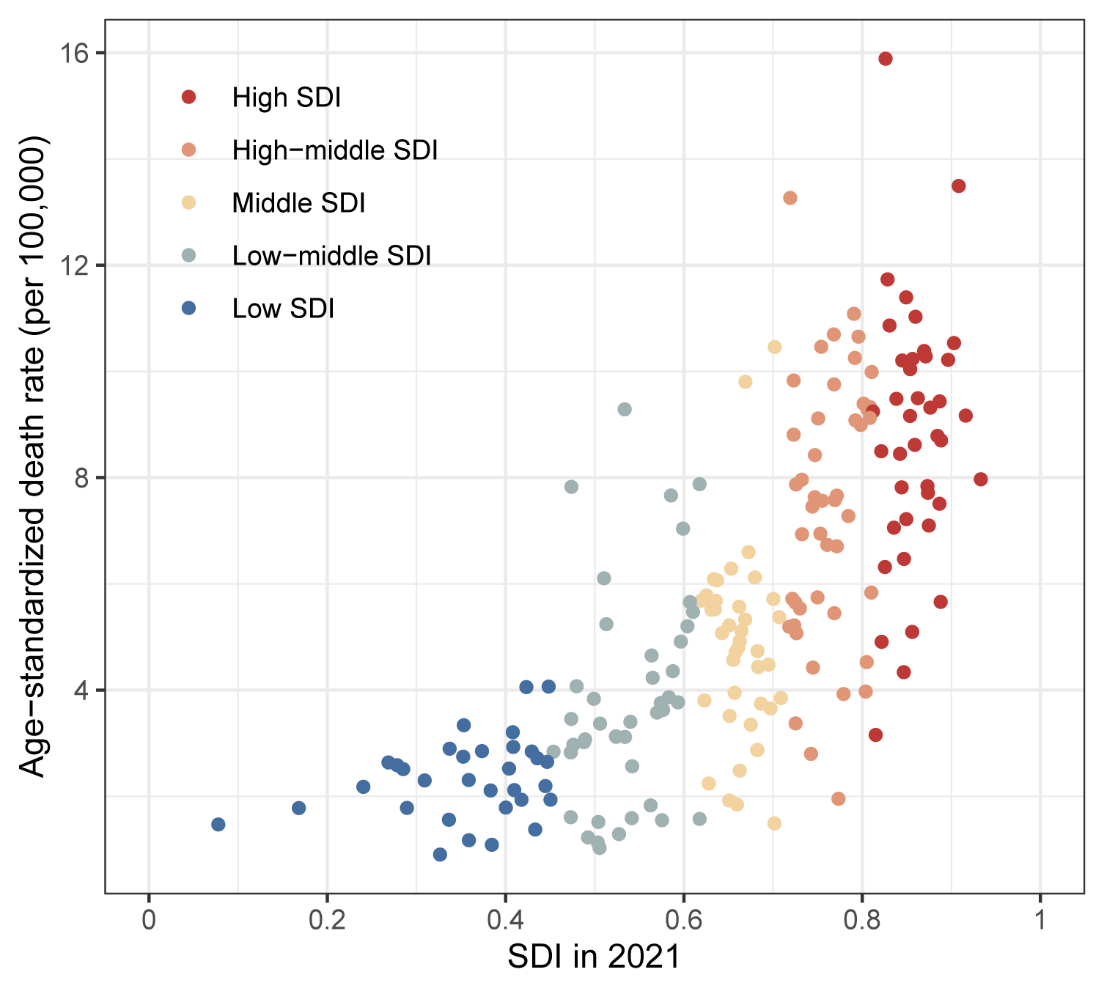
**

**Supplementary figure 7**

The decomposition analysis of pancreatic cancer DALYs change between 2019 and 2021 in different GBD regions. DALY: disability-adjusted-life-years; GBD: Global Burden of Disease

**
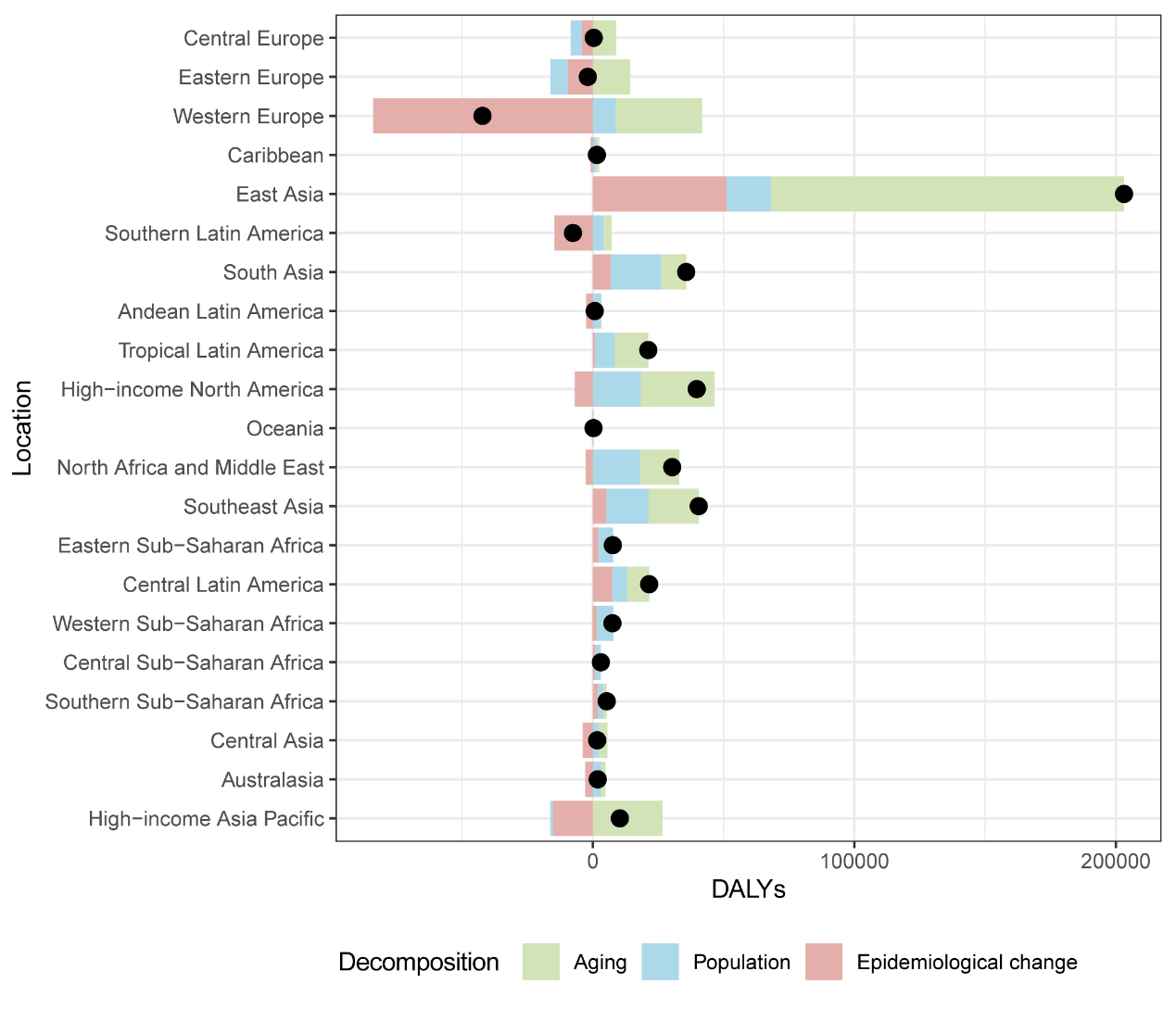
**
