# Supplementary material for: Global, regional and national burden of pancreatic cancer and its attributable risk factors from 2019 to 2021, with projection to 2044
Source: Front Oncol. 2025 Jan 14;14:1521788. doi: 10.3389/fonc.2024.1521788 (PMC11772166; doi:10.3389/fonc.2024.1521788)
Supplement: Supplementary file 2 [file Table1.docx]

**Supplementary table 1**

The incidence and death of pancreatic cancer in 2019 and 2021 at the national levels

| Location | ASIR in 2019 | ASDR in 2019 | ASIR in 2021 | ASDR in 2021 |
| --- | --- | --- | --- | --- |
| Afghanistan | 2.63 (1.67 to 4.5) | 2.82 (1.8 to 4.78) | 2.71 (1.74 to 4.64) | 2.9 (1.87 to 4.9) |
| Albania | 5.16 (3.77 to 6.8) | 5.55 (4.06 to 7.31) | 4.98 (3.48 to 6.76) | 5.37 (3.81 to 7.29) |
| Algeria | 1.72 (1.38 to 2.13) | 1.84 (1.48 to 2.26) | 1.73 (1.37 to 2.2) | 1.85 (1.47 to 2.33) |
| American Samoa | 4.87 (4.11 to 5.74) | 5.2 (4.39 to 6.14) | 4.89 (4.06 to 5.9) | 5.22 (4.34 to 6.29) |
| Andorra | 12.46 (9.56 to 15.89) | 12.54 (9.57 to 16.02) | 10.29 (7.05 to 13.61) | 10.39 (7.14 to 13.8) |
| Angola | 2.54 (1.56 to 3.69) | 2.75 (1.67 to 4.03) | 2.62 (1.59 to 3.77) | 2.84 (1.7 to 4.13) |
| Antigua and Barbuda | 5.58 (5.04 to 6.21) | 6.04 (5.45 to 6.71) | 5.32 (5.05 to 5.64) | 5.75 (5.44 to 6.1) |
| Argentina | 10.16 (9.49 to 10.83) | 10.84 (10.12 to 11.55) | 9.24 (8.51 to 9.9) | 9.83 (9.02 to 10.51) |
| Armenia | 10.01 (8.7 to 11.26) | 10.65 (9.25 to 11.99) | 9.82 (8.23 to 11.63) | 10.46 (8.68 to 12.32) |
| Australia | 9.09 (8.22 to 9.71) | 8.09 (7.32 to 8.64) | 8.76 (7.85 to 9.41) | 7.82 (7.05 to 8.38) |
| Austria | 11.17 (10.17 to 11.92) | 10.8 (9.79 to 11.53) | 10.37 (9.29 to 11.13) | 10.04 (9.01 to 10.8) |
| Azerbaijan | 4.53 (3.35 to 6.15) | 4.82 (3.57 to 6.45) | 4.21 (3.07 to 5.95) | 4.48 (3.25 to 6.32) |
| Bahamas | 4.32 (3.63 to 5.16) | 4.61 (3.88 to 5.48) | 4.24 (3.49 to 5.15) | 4.53 (3.73 to 5.49) |
| Bahrain | 6.46 (5.11 to 8.75) | 6.73 (5.35 to 9.05) | 6.67 (5.25 to 8.92) | 6.95 (5.44 to 9.26) |
| Bangladesh | 1.1 (0.75 to 1.55) | 1.19 (0.81 to 1.67) | 1.14 (0.78 to 1.61) | 1.23 (0.84 to 1.74) |
| Barbados | 7.66 (6.38 to 9) | 8.25 (6.88 to 9.64) | 7.08 (5.57 to 8.72) | 7.63 (6.01 to 9.31) |
| Belarus | 7.26 (6.68 to 7.96) | 7.26 (6.68 to 7.93) | 7.3 (5.99 to 8.85) | 7.28 (5.96 to 8.89) |
| Belgium | 9.44 (8.49 to 10.1) | 9.64 (8.62 to 10.37) | 8.98 (8.08 to 9.71) | 9.16 (8.16 to 9.9) |
| Belize | 5.33 (4.81 to 5.86) | 5.72 (5.14 to 6.27) | 5.11 (4.51 to 5.78) | 5.47 (4.84 to 6.16) |
| Benin | 2.53 (1.85 to 3.32) | 2.75 (2.04 to 3.6) | 2.62 (1.9 to 3.44) | 2.85 (2.09 to 3.75) |
| Bermuda | 8.14 (7.04 to 9.73) | 8.49 (7.32 to 10.09) | 8.16 (6.97 to 10.01) | 8.5 (7.22 to 10.35) |
| Bhutan | 1.45 (0.81 to 2.08) | 1.58 (0.88 to 2.27) | 1.48 (0.83 to 2.1) | 1.61 (0.91 to 2.28) |
| Bolivia (Plurinational State of) | 6.41 (4.28 to 8.83) | 7.03 (4.71 to 9.65) | 6.43 (4.29 to 9.04) | 7.04 (4.68 to 9.76) |
| Bosnia and Herzegovina | 8.82 (7.67 to 10.14) | 9.34 (8.11 to 10.75) | 8.3 (6.66 to 10.27) | 8.81 (7.05 to 10.89) |
| Botswana | 4.65 (3.41 to 6.02) | 5.08 (3.76 to 6.5) | 4.65 (3.34 to 6.17) | 5.07 (3.65 to 6.66) |
| Brazil | 5.94 (5.54 to 6.18) | 6.38 (5.92 to 6.65) | 5.88 (5.44 to 6.18) | 6.29 (5.78 to 6.62) |
| Brunei Darussalam | 6.16 (5.22 to 7.31) | 6.42 (5.44 to 7.59) | 5.63 (4.6 to 6.85) | 5.84 (4.75 to 7.08) |
| Bulgaria | 10.33 (9.31 to 11.45) | 10.7 (9.62 to 11.86) | 10.33 (8.68 to 12.09) | 10.7 (9.04 to 12.38) |
| Burkina Faso | 2.27 (1.62 to 3.03) | 2.48 (1.78 to 3.31) | 2.3 (1.65 to 3.13) | 2.51 (1.8 to 3.42) |
| Burundi | 1.64 (1.16 to 2.43) | 1.78 (1.25 to 2.66) | 1.64 (1.11 to 2.44) | 1.78 (1.2 to 2.68) |
| Cabo Verde | 8.51 (6.36 to 10.74) | 9.26 (6.9 to 11.67) | 8.55 (6.25 to 10.96) | 9.29 (6.73 to 11.86) |
| Cambodia | 3.16 (2.44 to 3.96) | 3.39 (2.62 to 4.25) | 3.23 (2.48 to 4.06) | 3.46 (2.67 to 4.35) |
| Cameroon | 3.7 (2.67 to 5.1) | 4.01 (2.92 to 5.5) | 3.75 (2.66 to 5.23) | 4.07 (2.9 to 5.7) |
| Canada | 9.78 (8.82 to 10.49) | 8.47 (7.7 to 9.04) | 9.05 (8.06 to 9.77) | 7.84 (6.99 to 8.47) |
| Central African Republic | 2.16 (1.59 to 2.91) | 2.33 (1.71 to 3.09) | 2.14 (1.56 to 2.9) | 2.3 (1.71 to 3.08) |
| Chad | 1.96 (1.51 to 2.46) | 2.13 (1.64 to 2.68) | 2 (1.52 to 2.56) | 2.18 (1.66 to 2.75) |
| Chile | 6.87 (6.33 to 7.37) | 7.2 (6.62 to 7.72) | 6.42 (5.89 to 6.94) | 6.71 (6.16 to 7.26) |
| China | 5.52 (4.55 to 6.61) | 5.62 (4.64 to 6.72) | 5.64 (4.52 to 6.84) | 5.72 (4.59 to 6.91) |
| Colombia | 4.1 (3.76 to 4.34) | 4.36 (3.98 to 4.63) | 4.31 (3.61 to 5.09) | 4.57 (3.78 to 5.37) |
| Comoros | 2.66 (1.93 to 3.61) | 2.89 (2.1 to 3.98) | 2.73 (1.97 to 3.73) | 2.97 (2.14 to 4.07) |
| Congo | 3.57 (2.35 to 5.05) | 3.86 (2.54 to 5.42) | 3.59 (2.37 to 5.14) | 3.87 (2.58 to 5.48) |
| Cook Islands | 3.77 (3.06 to 4.55) | 3.94 (3.21 to 4.74) | 3.76 (2.98 to 4.63) | 3.93 (3.11 to 4.8) |
| Costa Rica | 5.4 (4.92 to 5.82) | 5.75 (5.25 to 6.2) | 5.41 (4.79 to 6.11) | 5.72 (5.06 to 6.44) |
| C么te d'Ivoire | 1.68 (1.25 to 2.22) | 1.82 (1.37 to 2.41) | 1.71 (1.24 to 2.33) | 1.85 (1.36 to 2.52) |
| Croatia | 9.69 (8.8 to 10.67) | 9.12 (8.3 to 10.08) | 9.55 (8.29 to 10.98) | 8.99 (7.81 to 10.35) |
| Cuba | 5.18 (4.89 to 5.52) | 5.44 (5.13 to 5.79) | 5.07 (4.4 to 5.82) | 5.33 (4.62 to 6.16) |
| Cyprus | 7.55 (6.12 to 9.04) | 7.86 (6.41 to 9.41) | 6.78 (5.37 to 8.25) | 7.06 (5.57 to 8.61) |
| Czechia | 12.02 (10.72 to 13.2) | 12 (10.68 to 13.18) | 11.73 (9.97 to 13.47) | 11.73 (9.99 to 13.51) |
| Democratic People's Republic of Korea | 3.52 (2.33 to 4.7) | 3.63 (2.39 to 4.87) | 3.47 (2.22 to 4.67) | 3.58 (2.27 to 4.8) |
| Democratic Republic of the Congo | 1.9 (1.19 to 2.78) | 2.05 (1.28 to 3.08) | 1.95 (1.2 to 2.87) | 2.11 (1.28 to 3.18) |
| Denmark | 10.65 (9.7 to 11.39) | 10.64 (9.69 to 11.38) | 10.18 (9.24 to 10.97) | 10.22 (9.25 to 11.02) |
| Djibouti | 2.72 (1.7 to 3.93) | 2.96 (1.89 to 4.26) | 2.78 (1.75 to 4.05) | 3.02 (1.93 to 4.4) |
| Dominica | 7.73 (6.26 to 9.69) | 8.4 (6.77 to 10.44) | 7.75 (6.21 to 9.84) | 8.43 (6.76 to 10.77) |
| Dominican Republic | 5.15 (4.18 to 6.26) | 5.51 (4.46 to 6.72) | 5.3 (4.14 to 6.83) | 5.68 (4.44 to 7.44) |
| Ecuador | 4.61 (4.07 to 5.18) | 5.03 (4.44 to 5.66) | 4.41 (3.45 to 5.47) | 4.8 (3.78 to 5.93) |
| Egypt | 5.27 (4.77 to 5.81) | 5.55 (5.03 to 6.11) | 5.39 (4.48 to 6.55) | 5.66 (4.69 to 6.87) |
| El Salvador | 4.38 (3.91 to 4.97) | 4.63 (4.12 to 5.27) | 4.4 (3.56 to 5.43) | 4.65 (3.77 to 5.74) |
| Equatorial Guinea | 4.33 (2.49 to 6.47) | 4.68 (2.71 to 6.92) | 4.37 (2.54 to 6.23) | 4.71 (2.79 to 6.67) |
| Eritrea | 2.31 (1.45 to 3.2) | 2.51 (1.58 to 3.47) | 2.33 (1.46 to 3.27) | 2.52 (1.6 to 3.51) |
| Estonia | 10.56 (9.54 to 11.5) | 10.3 (9.29 to 11.22) | 10.47 (8.92 to 11.87) | 10.21 (8.67 to 11.61) |
| Eswatini | 7.48 (4.54 to 11.31) | 8.06 (4.96 to 12.11) | 7.16 (4.39 to 10.53) | 7.66 (4.76 to 11.28) |
| Ethiopia | 1.03 (0.61 to 1.63) | 1.12 (0.67 to 1.79) | 1.07 (0.64 to 1.68) | 1.17 (0.7 to 1.83) |
| Fiji | 3.07 (2.39 to 3.9) | 3.31 (2.59 to 4.16) | 3.1 (2.36 to 4.05) | 3.35 (2.54 to 4.29) |
| Finland | 12.53 (11.22 to 13.55) | 11.38 (10.2 to 12.29) | 12.15 (10.8 to 13.25) | 11.03 (9.84 to 12.01) |
| France | 11.08 (10.02 to 12.19) | 9.98 (9 to 10.98) | 10.51 (9.42 to 11.64) | 9.48 (8.46 to 10.52) |
| Gabon | 5.08 (3.53 to 6.75) | 5.5 (3.86 to 7.29) | 5.1 (3.64 to 6.71) | 5.52 (3.92 to 7.22) |
| Gambia | 1.96 (1.44 to 2.56) | 2.11 (1.53 to 2.8) | 1.96 (1.4 to 2.57) | 2.12 (1.49 to 2.84) |
| Georgia | 6.68 (5.98 to 7.45) | 7.03 (6.29 to 7.82) | 6.57 (5.72 to 7.53) | 6.94 (6.03 to 7.96) |
| Germany | 11.58 (10.64 to 12.35) | 10.96 (10.01 to 11.75) | 11.09 (10.12 to 11.9) | 10.54 (9.58 to 11.33) |
| Ghana | 3.82 (2.78 to 5.27) | 4.15 (3.02 to 5.75) | 3.89 (2.81 to 5.53) | 4.23 (3.06 to 6.02) |
| Greece | 9.91 (9.13 to 10.44) | 10.16 (9.35 to 10.7) | 10 (9.17 to 10.53) | 10.26 (9.41 to 10.82) |
| Greenland | 16.09 (13.36 to 19.18) | 16.83 (13.92 to 20.06) | 15.21 (12.4 to 18.51) | 15.89 (12.86 to 19.3) |
| Grenada | 9.2 (8.46 to 9.96) | 9.92 (9.15 to 10.75) | 9.1 (7.99 to 10.14) | 9.81 (8.6 to 10.91) |
| Guam | 4.66 (4.14 to 5.27) | 4.81 (4.27 to 5.44) | 3.9 (3.31 to 4.49) | 3.97 (3.35 to 4.58) |
| Guatemala | 3.31 (3.15 to 3.46) | 3.6 (3.41 to 3.75) | 3.16 (2.74 to 3.65) | 3.4 (2.94 to 3.91) |
| Guinea | 1.42 (1.07 to 1.83) | 1.54 (1.18 to 1.99) | 1.44 (1.08 to 1.93) | 1.56 (1.16 to 2.09) |
| Guinea-Bissau | 3.08 (2.22 to 4.03) | 3.33 (2.43 to 4.33) | 3.09 (2.18 to 4.07) | 3.34 (2.38 to 4.4) |
| Guyana | 4.86 (3.94 to 5.91) | 5.21 (4.23 to 6.31) | 4.88 (3.82 to 6.18) | 5.22 (4.1 to 6.57) |
| Haiti | 3.77 (2.64 to 5.2) | 4.11 (2.88 to 5.58) | 3.74 (2.58 to 5.14) | 4.07 (2.79 to 5.55) |
| Honduras | 4.83 (3.41 to 6.75) | 5.24 (3.71 to 7.33) | 4.84 (3.47 to 6.78) | 5.24 (3.74 to 7.34) |
| Hungary | 10.88 (9.88 to 11.86) | 11.4 (10.36 to 12.4) | 10.61 (9.19 to 12.06) | 11.09 (9.61 to 12.59) |
| Iceland | 9.62 (8.49 to 10.56) | 9.68 (8.53 to 10.65) | 9.29 (8.14 to 10.32) | 9.32 (8.15 to 10.39) |
| India | 1.43 (1.29 to 1.56) | 1.53 (1.39 to 1.68) | 1.45 (1.26 to 1.62) | 1.55 (1.35 to 1.74) |
| Indonesia | 3.63 (2.61 to 4.65) | 3.89 (2.79 to 5) | 3.68 (2.63 to 4.71) | 3.95 (2.8 to 5.08) |
| Iran (Islamic Republic of) | 3.82 (3.41 to 4.16) | 3.91 (3.48 to 4.26) | 3.57 (3.14 to 3.96) | 3.65 (3.21 to 4.04) |
| Iraq | 4.41 (3.33 to 5.63) | 4.57 (3.47 to 5.77) | 4.75 (3.52 to 6.05) | 4.93 (3.66 to 6.24) |
| Ireland | 8.73 (7.89 to 9.47) | 8.85 (7.98 to 9.61) | 7.61 (6.83 to 8.4) | 7.71 (6.88 to 8.52) |
| Israel | 9.72 (8.8 to 10.44) | 10.09 (9.11 to 10.85) | 8.97 (7.94 to 9.71) | 9.33 (8.22 to 10.12) |
| Italy | 9.74 (8.68 to 10.4) | 9.52 (8.45 to 10.19) | 9.48 (8.44 to 10.16) | 9.29 (8.23 to 9.97) |
| Jamaica | 4.18 (3.43 to 5.11) | 4.47 (3.68 to 5.45) | 4.15 (3.25 to 5.35) | 4.43 (3.48 to 5.62) |
| Japan | 11.71 (10.29 to 12.5) | 10.42 (9.22 to 11.11) | 11.55 (10.13 to 12.34) | 10.28 (9.08 to 10.97) |
| Jordan | 3.2 (2.46 to 4.29) | 3.3 (2.52 to 4.44) | 3.28 (2.43 to 4.37) | 3.37 (2.5 to 4.58) |
| Kazakhstan | 5.52 (4.78 to 6.3) | 5.79 (5 to 6.58) | 5.38 (4.56 to 6.26) | 5.65 (4.79 to 6.54) |
| Kenya | 2.82 (2.24 to 3.63) | 3.06 (2.43 to 3.94) | 2.89 (2.26 to 3.69) | 3.13 (2.45 to 4) |
| Kiribati | 1.17 (0.87 to 1.56) | 1.28 (0.95 to 1.71) | 1.18 (0.85 to 1.58) | 1.29 (0.94 to 1.73) |
| Kuwait | 4.34 (3.89 to 4.72) | 4.3 (3.85 to 4.67) | 4.39 (3.58 to 5.31) | 4.34 (3.57 to 5.28) |
| Kyrgyzstan | 5.03 (4.38 to 5.77) | 5.32 (4.62 to 6.11) | 4.93 (4.02 to 6.04) | 5.2 (4.28 to 6.33) |
| Lao People's Democratic Republic | 2.83 (2.06 to 3.77) | 3.03 (2.23 to 4.01) | 2.87 (2.08 to 3.8) | 3.07 (2.25 to 4.05) |
| Latvia | 10.63 (9.53 to 11.81) | 10.85 (9.72 to 12.02) | 10.67 (9.12 to 12.43) | 10.87 (9.3 to 12.58) |
| Lebanon | 4.37 (3.25 to 5.83) | 4.47 (3.31 to 5.95) | 4.33 (3.16 to 5.85) | 4.42 (3.23 to 5.97) |
| Lesotho | 5.84 (3.87 to 8.15) | 6.31 (4.26 to 8.81) | 5.68 (3.7 to 8.1) | 6.11 (4.02 to 8.71) |
| Liberia | 2.47 (1.45 to 3.74) | 2.68 (1.58 to 4.08) | 2.53 (1.43 to 3.82) | 2.75 (1.57 to 4.16) |
| Libya | 7.73 (5.45 to 9.95) | 7.99 (5.63 to 10.28) | 7.59 (5.36 to 10.01) | 7.87 (5.61 to 10.4) |
| Lithuania | 9.88 (9.16 to 10.62) | 10.23 (9.5 to 11) | 9.9 (8.6 to 11.12) | 10.24 (8.93 to 11.52) |
| Luxembourg | 9.38 (8.6 to 10.21) | 9.62 (8.81 to 10.49) | 8.58 (7.67 to 9.43) | 8.79 (7.87 to 9.69) |
| Madagascar | 1.62 (1.14 to 2.24) | 1.76 (1.23 to 2.41) | 1.65 (1.13 to 2.31) | 1.79 (1.22 to 2.49) |
| Malawi | 0.99 (0.73 to 1.32) | 1.07 (0.79 to 1.43) | 1 (0.72 to 1.37) | 1.09 (0.78 to 1.47) |
| Malaysia | 2.66 (2.27 to 3.05) | 2.83 (2.4 to 3.25) | 2.63 (2.24 to 3.05) | 2.8 (2.37 to 3.26) |
| Maldives | 1.84 (1.52 to 2.24) | 1.97 (1.64 to 2.4) | 1.79 (1.41 to 2.18) | 1.92 (1.52 to 2.34) |
| Mali | 2.43 (1.88 to 3.1) | 2.64 (2.03 to 3.38) | 2.42 (1.82 to 3.13) | 2.64 (1.99 to 3.42) |
| Malta | 9.25 (8.26 to 10.23) | 9.41 (8.37 to 10.4) | 9.24 (8.22 to 10.35) | 9.39 (8.34 to 10.55) |
| Marshall Islands | 3.47 (2.57 to 4.68) | 3.72 (2.77 to 4.98) | 3.51 (2.56 to 4.75) | 3.76 (2.77 to 5.07) |
| Mauritania | 3.33 (2.45 to 4.34) | 3.62 (2.69 to 4.72) | 3.53 (2.55 to 4.66) | 3.84 (2.79 to 5.11) |
| Mauritius | 4.95 (4.65 to 5.22) | 5.18 (4.86 to 5.46) | 4.97 (4.62 to 5.23) | 5.19 (4.81 to 5.46) |
| Mexico | 4.71 (4.54 to 4.83) | 5.04 (4.85 to 5.17) | 4.8 (4.23 to 5.36) | 5.12 (4.53 to 5.71) |
| Micronesia (Federated States of) | 4.01 (2.9 to 5.24) | 4.3 (3.11 to 5.62) | 4.06 (2.88 to 5.35) | 4.36 (3.1 to 5.78) |
| Monaco | 13.33 (8.83 to 19.53) | 13.55 (8.93 to 19.91) | 13.27 (8.78 to 19.3) | 13.49 (8.88 to 19.65) |
| Mongolia | 7.92 (5.79 to 10.48) | 8.39 (6.13 to 11.17) | 7.4 (5.46 to 9.86) | 7.88 (5.86 to 10.51) |
| Montenegro | 10.36 (8.42 to 12.29) | 10.95 (8.91 to 13.06) | 10.02 (7.92 to 12.26) | 10.66 (8.45 to 12.98) |
| Morocco | 1.71 (1.28 to 2.1) | 1.8 (1.35 to 2.21) | 1.74 (1.29 to 2.12) | 1.83 (1.36 to 2.23) |
| Mozambique | 0.81 (0.62 to 1.03) | 0.91 (0.7 to 1.16) | 0.81 (0.61 to 1.04) | 0.9 (0.68 to 1.17) |
| Myanmar | 2.85 (2.17 to 3.67) | 3.05 (2.33 to 3.91) | 2.91 (2.19 to 3.79) | 3.12 (2.36 to 4.07) |
| Namibia | 1.48 (1.09 to 1.85) | 1.58 (1.18 to 1.99) | 1.47 (1.07 to 1.88) | 1.57 (1.16 to 2.01) |
| Nauru | 5.34 (3.4 to 7.24) | 5.7 (3.64 to 7.76) | 5.43 (3.37 to 7.45) | 5.78 (3.61 to 7.9) |
| Nepal | 1.23 (0.73 to 1.84) | 1.34 (0.79 to 1.99) | 1.27 (0.76 to 1.88) | 1.37 (0.82 to 2.06) |
| Netherlands | 8.28 (7.54 to 8.8) | 9.06 (8.19 to 9.66) | 7.91 (7.1 to 8.48) | 8.7 (7.82 to 9.37) |
| New Zealand | 8.15 (7.4 to 8.79) | 7.46 (6.83 to 8.05) | 7.89 (7.09 to 8.49) | 7.22 (6.49 to 7.8) |
| Nicaragua | 3.1 (2.65 to 3.57) | 3.32 (2.83 to 3.81) | 2.93 (2.32 to 3.63) | 3.12 (2.48 to 3.86) |
| Niger | 1.6 (1.05 to 2.2) | 1.75 (1.15 to 2.43) | 1.62 (1.07 to 2.3) | 1.78 (1.16 to 2.51) |
| Nigeria | 1 (0.78 to 1.26) | 1.1 (0.86 to 1.38) | 1.03 (0.79 to 1.31) | 1.13 (0.89 to 1.42) |
| Niue | 4.63 (3.57 to 5.93) | 4.97 (3.86 to 6.29) | 4.74 (3.62 to 6.11) | 5.07 (3.88 to 6.53) |
| North Macedonia | 8.96 (7.42 to 10.67) | 9.69 (8.03 to 11.6) | 8.46 (6.52 to 10.77) | 9.12 (7.11 to 11.57) |
| Northern Mariana Islands | 6.99 (5.72 to 8.35) | 7.46 (6.1 to 8.93) | 7.17 (6.01 to 8.43) | 7.66 (6.42 to 9.03) |
| Norway | 10.11 (9.23 to 10.69) | 9.52 (8.68 to 10.06) | 9.71 (8.79 to 10.29) | 9.17 (8.32 to 9.72) |
| Oman | 2.14 (1.68 to 2.67) | 2.17 (1.71 to 2.67) | 1.92 (1.42 to 2.41) | 1.95 (1.45 to 2.44) |
| Pakistan | 1.37 (1.07 to 1.79) | 1.49 (1.16 to 1.94) | 1.4 (1.09 to 1.89) | 1.52 (1.19 to 2.05) |
| Palau | 9.57 (7.76 to 11.75) | 10.61 (8.55 to 13.01) | 9.44 (7.57 to 11.53) | 10.46 (8.4 to 12.93) |
| Palestine | 5.11 (4.31 to 5.9) | 5.39 (4.57 to 6.25) | 5.24 (4.33 to 6.19) | 5.51 (4.55 to 6.52) |
| Panama | 3.58 (3.33 to 3.8) | 3.85 (3.58 to 4.09) | 3.6 (2.85 to 4.29) | 3.85 (3.04 to 4.59) |
| Papua New Guinea | 1.78 (1.28 to 2.58) | 1.92 (1.37 to 2.83) | 1.79 (1.24 to 2.61) | 1.93 (1.34 to 2.85) |
| Paraguay | 5.49 (4.42 to 6.73) | 5.96 (4.78 to 7.25) | 5.26 (4 to 6.85) | 5.68 (4.32 to 7.34) |
| Peru | 5.5 (4.3 to 6.87) | 5.89 (4.62 to 7.29) | 5.22 (3.83 to 6.9) | 5.57 (4.09 to 7.35) |
| Philippines | 3.29 (2.95 to 3.64) | 3.49 (3.13 to 3.87) | 3.31 (2.78 to 3.93) | 3.51 (2.95 to 4.16) |
| Poland | 8.5 (8.06 to 8.82) | 9.26 (8.76 to 9.62) | 8.47 (7.72 to 9.19) | 9.25 (8.43 to 10.01) |
| Portugal | 7.25 (6.63 to 7.76) | 7.78 (7.1 to 8.35) | 6.93 (6.28 to 7.45) | 7.46 (6.73 to 8.04) |
| Puerto Rico | 6.06 (5.6 to 6.5) | 6.28 (5.78 to 6.72) | 6.1 (5.02 to 7.2) | 6.32 (5.22 to 7.44) |
| Qatar | 6.11 (4.5 to 8.47) | 6.13 (4.56 to 8.49) | 6.4 (4.7 to 9) | 6.47 (4.77 to 9.1) |
| Republic of Korea | 8.25 (6.63 to 9.85) | 7.54 (6.12 to 8.98) | 8.23 (6.45 to 9.99) | 7.51 (5.88 to 9.14) |
| Republic of Moldova | 7.77 (7.05 to 8.45) | 7.88 (7.14 to 8.56) | 7.85 (6.97 to 8.8) | 7.96 (7.06 to 8.91) |
| Romania | 9.2 (8.37 to 9.92) | 9.6 (8.73 to 10.35) | 9.35 (8.12 to 10.6) | 9.76 (8.49 to 11.08) |
| Russian Federation | 8.8 (8.47 to 9.05) | 9.14 (8.79 to 9.42) | 8.78 (8.07 to 9.5) | 9.12 (8.4 to 9.88) |
| Rwanda | 2.39 (1.7 to 3.43) | 2.62 (1.85 to 3.76) | 2.49 (1.72 to 3.59) | 2.72 (1.87 to 3.96) |
| Saint Kitts and Nevis | 6.9 (5.8 to 7.84) | 7.54 (6.36 to 8.53) | 6.93 (5.81 to 7.99) | 7.57 (6.31 to 8.66) |
| Saint Lucia | 5.78 (5.01 to 6.67) | 6.25 (5.4 to 7.17) | 6.08 (5.04 to 7.32) | 6.6 (5.44 to 7.91) |
| Saint Vincent and the Grenadines | 5.62 (5.14 to 6.2) | 6.07 (5.53 to 6.69) | 5.63 (4.99 to 6.4) | 6.07 (5.38 to 6.87) |
| Samoa | 3.5 (2.8 to 4.35) | 3.75 (3.01 to 4.63) | 3.52 (2.8 to 4.43) | 3.77 (3 to 4.76) |
| San Marino | 8.66 (6.94 to 10.71) | 8.82 (7.08 to 10.9) | 5.59 (3.61 to 8.02) | 5.66 (3.67 to 8.09) |
| Sao Tome and Principe | 0.94 (0.74 to 1.27) | 1.03 (0.8 to 1.38) | 0.95 (0.73 to 1.29) | 1.03 (0.8 to 1.38) |
| Saudi Arabia | 3.15 (2.64 to 3.85) | 3.22 (2.7 to 3.94) | 3.09 (2.51 to 3.83) | 3.16 (2.54 to 3.92) |
| Senegal | 2.88 (2.19 to 3.65) | 3.14 (2.39 to 3.99) | 2.94 (2.24 to 3.77) | 3.21 (2.45 to 4.11) |
| Serbia | 8.46 (7 to 10.11) | 9.05 (7.47 to 10.87) | 8.49 (6.67 to 10.46) | 9.08 (7.15 to 11.13) |
| Seychelles | 6.02 (5.13 to 6.88) | 6.37 (5.42 to 7.29) | 5.22 (4.35 to 6.2) | 5.54 (4.6 to 6.56) |
| Sierra Leone | 2.09 (1.55 to 2.69) | 2.27 (1.69 to 2.95) | 2.12 (1.54 to 2.76) | 2.31 (1.68 to 3.02) |
| Singapore | 5.93 (5.35 to 6.37) | 5.46 (4.95 to 5.87) | 5.54 (4.94 to 5.98) | 5.1 (4.6 to 5.5) |
| Slovakia | 9.72 (8.04 to 11.7) | 10 (8.31 to 12.04) | 9.69 (7.69 to 12.29) | 9.99 (7.93 to 12.58) |
| Slovenia | 9.01 (8.16 to 9.79) | 8.99 (8.16 to 9.76) | 8.45 (7.15 to 9.72) | 8.45 (7.13 to 9.73) |
| Solomon Islands | 2.65 (1.98 to 3.56) | 2.83 (2.13 to 3.83) | 2.66 (1.96 to 3.58) | 2.84 (2.12 to 3.84) |
| Somalia | 1.39 (0.72 to 2.31) | 1.5 (0.77 to 2.52) | 1.36 (0.7 to 2.25) | 1.47 (0.75 to 2.45) |
| South Africa | 5.53 (4.94 to 6.12) | 5.98 (5.36 to 6.62) | 5.67 (4.98 to 6.33) | 6.12 (5.38 to 6.84) |
| South Sudan | 2.38 (1.43 to 3.4) | 2.57 (1.52 to 3.68) | 2.39 (1.45 to 3.39) | 2.58 (1.55 to 3.69) |
| Spain | 8.34 (7.49 to 8.95) | 8 (7.19 to 8.58) | 7.89 (6.96 to 8.54) | 7.58 (6.68 to 8.19) |
| Sri Lanka | 1.49 (1.08 to 2.03) | 1.56 (1.13 to 2.12) | 1.43 (0.93 to 1.97) | 1.49 (0.98 to 2.07) |
| Sudan | 2.34 (1.55 to 3.53) | 2.48 (1.66 to 3.74) | 2.42 (1.57 to 3.67) | 2.56 (1.66 to 3.95) |
| Suriname | 6.36 (5.43 to 7.29) | 6.82 (5.8 to 7.8) | 5.69 (4.34 to 7.35) | 6.09 (4.61 to 7.88) |
| Sweden | 9.55 (8.67 to 10.21) | 10.43 (9.44 to 11.19) | 8.59 (7.46 to 9.6) | 9.44 (8.2 to 10.57) |
| Switzerland | 8.85 (7.88 to 9.73) | 8.44 (7.5 to 9.32) | 8.3 (7.36 to 9.19) | 7.97 (7.03 to 8.86) |
| Syrian Arab Republic | 3.68 (2.67 to 4.82) | 3.83 (2.8 to 4.95) | 3.66 (2.63 to 4.91) | 3.8 (2.73 to 5.11) |
| Tajikistan | 1.45 (0.99 to 1.98) | 1.57 (1.07 to 2.13) | 1.47 (1.02 to 2.08) | 1.59 (1.11 to 2.24) |
| Thailand | 4.49 (3.82 to 5.32) | 4.63 (3.95 to 5.48) | 4.59 (3.57 to 5.91) | 4.73 (3.69 to 6.03) |
| Timor-Leste | 2.05 (1.63 to 2.59) | 2.21 (1.76 to 2.8) | 2.04 (1.58 to 2.57) | 2.19 (1.71 to 2.79) |
| Togo | 2.63 (1.84 to 3.54) | 2.85 (1.99 to 3.85) | 2.7 (1.86 to 3.68) | 2.93 (2.01 to 3.99) |
| Tokelau | 3.45 (2.42 to 4.68) | 3.7 (2.61 to 4.99) | 3.5 (2.46 to 4.74) | 3.74 (2.65 to 5.06) |
| Tonga | 5.29 (4.07 to 6.75) | 5.66 (4.37 to 7.23) | 5.33 (4.04 to 6.87) | 5.7 (4.35 to 7.27) |
| Trinidad and Tobago | 5.08 (4.35 to 5.91) | 5.42 (4.64 to 6.32) | 5.1 (3.88 to 6.46) | 5.45 (4.15 to 6.87) |
| Tunisia | 2.79 (2.02 to 3.74) | 2.88 (2.07 to 3.85) | 2.8 (2.02 to 3.8) | 2.87 (2.09 to 3.88) |
| T眉rkiye | 7.88 (6.66 to 9.25) | 8.11 (6.87 to 9.51) | 7.98 (6.22 to 9.61) | 8.19 (6.44 to 9.86) |
| Turkmenistan | 2.74 (2.14 to 3.5) | 2.88 (2.26 to 3.68) | 2.73 (2.08 to 3.63) | 2.87 (2.2 to 3.81) |
| Tuvalu | 3.33 (2.61 to 4.17) | 3.58 (2.8 to 4.49) | 3.38 (2.63 to 4.29) | 3.63 (2.8 to 4.62) |
| Uganda | 3.65 (2.7 to 4.84) | 3.96 (2.95 to 5.23) | 3.73 (2.69 to 5.06) | 4.06 (2.92 to 5.54) |
| Ukraine | 7.13 (6.05 to 8.47) | 7.15 (6.08 to 8.48) | 6.73 (5.01 to 8.58) | 6.73 (5.04 to 8.6) |
| United Arab Emirates | 15.57 (12.35 to 19.11) | 16.7 (13.25 to 20.56) | 10.65 (8.52 to 13.15) | 11.4 (9.11 to 14.14) |
| United Kingdom | 9.18 (8.46 to 9.54) | 9.12 (8.39 to 9.49) | 8.67 (7.97 to 9.02) | 8.62 (7.92 to 8.97) |
| United Republic of Tanzania | 2.38 (1.71 to 3.33) | 2.58 (1.85 to 3.65) | 2.44 (1.78 to 3.52) | 2.65 (1.92 to 3.82) |
| United States of America | 10.28 (9.48 to 10.73) | 9.46 (8.68 to 9.88) | 10.33 (9.53 to 10.8) | 9.5 (8.74 to 9.95) |
| United States Virgin Islands | 5.52 (4.25 to 7.13) | 5.9 (4.55 to 7.55) | 4.59 (3.45 to 6.03) | 4.91 (3.67 to 6.41) |
| Uruguay | 12.63 (11.62 to 13.49) | 13.38 (12.31 to 14.3) | 12.55 (11.38 to 13.72) | 13.27 (12.06 to 14.54) |
| Uzbekistan | 2.37 (2.04 to 2.74) | 2.5 (2.15 to 2.9) | 2.35 (1.87 to 2.86) | 2.48 (1.98 to 3.02) |
| Vanuatu | 2.59 (2.13 to 3.15) | 2.79 (2.3 to 3.38) | 2.62 (2.13 to 3.21) | 2.83 (2.29 to 3.48) |
| Venezuela (Bolivarian Republic of) | 4.51 (3.57 to 5.59) | 4.82 (3.83 to 5.99) | 4.6 (3.34 to 5.97) | 4.91 (3.58 to 6.38) |
| Viet Nam | 2.11 (1.64 to 2.55) | 2.22 (1.74 to 2.69) | 2.13 (1.6 to 2.59) | 2.24 (1.69 to 2.73) |
| Yemen | 1.79 (1.17 to 2.54) | 1.91 (1.27 to 2.71) | 1.81 (1.16 to 2.51) | 1.94 (1.25 to 2.68) |
| Zambia | 3.1 (2.26 to 4.07) | 3.35 (2.47 to 4.36) | 3.12 (2.24 to 4.14) | 3.37 (2.44 to 4.46) |
| Zimbabwe | 7.36 (5.72 to 9.21) | 7.98 (6.22 to 10.01) | 7.25 (5.54 to 9.16) | 7.83 (5.98 to 9.82) |

ASIR: age-standardized incidence rate; ASDR: age-standardized death rate
